# COVID–19: A Surgical Perspective for when the curve flattens

**Published:** 2020-08-05

**Authors:** GN Moawad, JS Klebanoff, P Tyan, JI Einarsson

**Affiliations:** Department of Obstetrics and Gynecology, The George Washington University, Washington, DC;; Division of Minimally Invasive Gynecologic Surgery, Department of Obstetrics and Gynecology, The University of North Carolina, Chapel Hill, NC;; Division of Minimally Invasive Gynecologic Surgery Department of Obstetrics and Gynecology, Brigham and Women’s Hospital, Boston, MA.

**Keywords:** COVID-19, surgery, flatten the curve, response

## Abstract

At the present time it is clear that our global healthcare community was not prepared to face the COVID-19 pandemic. Hospitals in the hardest hit areas have been transformed to COVID centres. Surgical societies have recommended postponing non-emergency surgery, and have given recommendations for triaging the ever- growing backlog of patients. However, simply resuming these non-emergency surgeries may lead the healthcare system into a second disaster. If healthcare policymakers around the world do not systematically consider how to resume normal surgical services, hospitals will be quickly overwhelmed, vital resources will be depleted, and patients and providers alike will face an increased exposure risk. This perspective serves to highlight certain aspects of returning to normal that physicians and hospital administrators alike must consider to avoid potential catastrophe.

## Perspective

Corona Virus Disease-19 (COVID-19), the illness caused by the novel coronavirus, SARS-CoV-2, has disrupted global healthcare delivery so much so that caring for patients may never be the same. Moreover, global economies are in turmoil – supply chains are frozen, vital resources for both healthcare delivery and everyday life have become dangerously scarce, and unemployment claims are unprecedented, as businesses remain shuttered. As death rates climb, much of our focus, rightfully so, has centred on present-day issues: Are we flattening the curve? How long will vital healthcare resources last? When can we resume our normal lives? However, there remains a largely unaddressed and equally important question: Are we prepared for healthcare delivery after we flatten the curve?

It is indisputable that global healthcare systems are poorly prepared to face this pandemic. Retirees are reentering the workforce, ungraduated medical students are being sent to the front lines, and physicians are practicing in areas of medicine outside of their specialty – we are in a crisis (Levy, 2020). Many healthcare systems have been left to fend for themselves as available numbers of life- saving ventilators dwindle, and Personal Protective Equipment (PPE) supplies wane.

Many surgical societies and the Centers for Disease Control and Prevention have published recommendations to postpone elective surgeries during the COVID-19 pandemic (Centers for Disease Control and Prevention, 2020). Those recommendations have been put in place to minimise unnecessary contact between patients and medical staff, and to conserve vital resources. By doing so, countless lives have been impacted. Annually an estimated 50 million elective surgical procedures are performed in the United States alone - hundreds of millions on a global scale (Hall et al., 2017). COVID-19 has reshaped the current healthcare delivery landscape. Hospitals will not be ready to cope with the volume once elective procedures are reinstated. We will have survived a global crisis only to face yet another, and this time it will be of our own doing. We missed the opportunity to adequately prepare for the COVID-19 pandemic; we should do so now for this new potential crisis.

Existing patients have been rescheduled, and providers are currently adding to their backlog of postponed surgeries via telehealth. The first preparatory step towards optimising hospital capabilities will be to maintain a triaging system for elective surgeries once restrictions are lifted. Such systems need to be implemented to determine the urgency of any given procedure based on the weighted assessment of morbidity associated with continued delay. We cannot know what ramifications our actions will have by perpetuating both benign and malignant diseases. Not all surgeries during the COVID-19 pandemic were cancelled equally; determinations must be made on which procedures can safely be delayed and which cannot.

Surgical societies must come together and develop standardised metrics by which surgeons and hospitals can determine how to reschedule procedures. Metrics to be taken into consideration should include the morbidity associated with the primary diagnosis, the projected morbidity caused by the delay, the underlying health of the patient and likelihood of poor perioperative outcomes, the risk of COVID-19 exposure to both patients and healthcare workers, available resources in a geographic location, and the impact of the procedure on those resources. Healthcare resources will be in an extremely vulnerable position, as the reinstatement of elective surgery will not necessarily coincide with the return of normal supply chain function. Hospital inventories must be considered, factoring in potential delays on needed surgical equipment as this wave of demand hits a crippled supply. Surgeons must be prepared to use unfamiliar equipment, circumvent medication shortages, anticipate delays in accessing the operating room, and minimise their strain on their institution. Hospitals may increase operating room access by extending surgical hours during weekdays and consider performing elective procedures on weekends. To ensure the safety of all non-COVID patients and staff, hospital leadership should consider section division within their institution. Hospital systems with more than one location should consider a COVID-19 dedicated facility to keep the other sites COVID-19-free. COVID-19 positive patients will continue to need hospitalisation, and we must keep healthy patients and staff separated from contact with the virus. Separate closed units, operating rooms, hospital entrances, and resource storage should be created so that we work to minimise cross-contamination.

Given the asymptomatic carrier profile of the disease, we must ensure the safety of our patients and our providers during this time (Sutton et al., 2020). Patients should be tested upon arrival, and if positive, non-urgent elective cases should be postponed until the patient has recovered from the infection. Since there is a chance of a false negative test, providers should still use appropriate PPE during the procedure, and filters should be used during laparoscopic surgery. Once antibody testing is widely available and the immune response to the virus is better elucidated, patients can be tested for antibodies at their preoperative visit. Seroconverted patients can proceed with surgery without further testing.

Next, we must focus on how to minimise hospital resource utilisation. Surgeons should reduce strain on hospitals by increasing their utilisation of Ambulatory Surgery Centres (ACS) when possible. Outpatient procedures performed in ACS will not affect hospital resources. Ample evidence supports the excellent outcomes following ambulatory surgery. In the United States (US), nearly 50% of outpatient surgical procedures are performed in ACS (Hall et al., 2017). This percentage must increase to allow hospital resources use to be dedicated to the most critically ill patients. All healthcare institutions, ACS included, are under severe financial strains. As we reinstate elective procedures, institutional leadership should consider and avoid preferentially rescheduling procedures with high-profit margins over less profitable ones, at least in settings without nationalised medicine.

Lastly, we suggest that surgical procedures during this transition period be performed by subspecialty- trained or high-volume surgeons. Data from a large number of publications highlight the improved patient outcomes and lower healthcare resource utilisation when procedures are performed by high-volume minimally invasive surgeons (Akhtar- Danesh et al., 2018). However, the shortcomings of volume as the singular surgical metric have perhaps never been so important. We must not only rely on a simple numbers game and focus our attention on patient outcomes. Surgeons must make every effort to perform minimally invasive surgery whenever feasible to minimise further strain on an already burdened healthcare system and minimise hospital length of stay for patients. Physicians should seek out referral surgeons to complete minimally invasive surgery if unable to offer this surgical route themselves. Such a practice shift will decrease complication rates, decrease hospital length of stay, lower laparotomy conversion rates, hospital readmission rates, and precious resource utilisation. To achieve this goal, our surgical societies must partner with the Centers for Medicare and Medicaid Services, and consider restructuring reimbursements for surgical services based on predetermined surgical metrics, such as surgeon volume or training.

Recovering fully from COVID-19 is going to be challenging and will likely require a unified global effort. Full recovery is undoubtedly going to be a slow process, and as we try to return to normality we must minimise the potential for a second wave of disease. We must learn from our mistakes for a stronger recovery, and be better prepared for what lies ahead. We can better position ourselves by creatively triaging our postponed non-emergency surgeries, increasing utilisation of ACS or considering sectioning of hospitals, developing metrics that consider not only surgeon volume but perioperative outcomes, and then funneling the bulk of necessary surgeries to these surgeons. Surgeons must advocate for their institutions, their patients, and themselves. Creative thinking is needed to ensure that we do not come out of one catastrophe only to learn that our actions have created another.
